# Airway inflammation contributes to health status in COPD: a cross-sectional study

**DOI:** 10.1186/1465-9921-7-140

**Published:** 2006-11-30

**Authors:** Jiska B Snoeck-Stroband, Dirkje S Postma, Thérèse S Lapperre, Margot ME Gosman, Henk A Thiadens, Henk F Kauffman, Jacob K Sont, Désirée F Jansen, Peter J Sterk

**Affiliations:** 1 General Practice, Leiden University Medical Center, Leiden, The Netherlands; 2 Pulmonology, Leiden University Medical Center, Leiden, The Netherlands; 3 Medical Decision Making, Leiden University Medical Center, Leiden, The Netherlands; 4 Pulmonology, University Medical Center Groningen, Groningen, The Netherlands; 5 Allergology, University Medical Center Groningen, Groningen, The Netherlands; 6 Epidemiology and Bioinformatics, University Medical Center Groningen, Groningen, The Netherlands

## Abstract

**Background:**

Chronic obstructive pulmonary disease (COPD) is characterized by irreversible airflow limitation and airway inflammation, accompanied by decreased health status. It is still unknown which factors are responsible for the impaired health status in COPD. We postulated that airway inflammation negatively contributes to health status in COPD.

**Methods:**

In 114 COPD patients (99 male, age: 62 ± 8 yr, 41 [31–55] pack-years, no inhaled or oral corticosteroids, postbronchodilator FEV_1_: 63 ± 9% pred, FEV_1_/IVC: 48 ± 9%) we obtained induced sputum and measured health status (St. George's respiratory questionnaire (SGRQ)), postbronchodilator FEV_1_, hyperinflation (RV/TLC), and airway hyperresponsiveness to methacholine (PC_20_). Sputum was induced by hypertonic saline and differential cell counts were obtained in 102 patients.

**Results:**

Univariate analysis showed that SGRQ total and symptom score were positively associated with % sputum macrophages (r = 0.20, p = 0.05; and r = 0.20, p = 0.04, respectively). Multiple regression analysis confirmed these relationships, providing significant contributions of % sputum macrophages (B = 0.25, p = 0.021) and RV/TLC (B = 0.60, p = 0.002) to SGRQ total score. Furthermore, SGRQ symptom score was associated with % sputum macrophages (B = 0.30, p = 0.03) and RV/TLC (B = 0.48, p = 0.044), whilst SGRQ activity score was associated with % sputum macrophages (B = 0.46, p = 0.002), RV/TLC (B = 0.61, p = 0.015), and PC_20 _(B = -9.3, p = 0.024). Current smoking and FEV_1 _were not significantly associated with health status in the multiple regression analysis.

**Conclusion:**

We conclude that worse health status in COPD patients is associated with higher inflammatory cell counts in induced sputum. Our findings suggest that airway inflammation and hyperinflation independently contribute to impaired health status in COPD. This may provide a rationale for anti-inflammatory therapy in this disease.

## Background

Chronic obstructive pulmonary disease (COPD) is a major and growing cause of morbidity and mortality [1,2]. It is characterized by progressive and not fully reversible airflow limitation, as measured with the forced expiratory volume in one second (FEV_1_). The airflow limitation is associated with a chronic inflammatory process in the airways and lung parenchyma in response to noxious particles or gases, in particular tobacco smoking [1,2].

In daily life COPD patients are bothered by airway symptoms such as dyspnea, cough and sputum production [2,3]. This is accompanied by a serious decrease of health status [4]. Several studies have attempted to link health status to the severity of airflow limitation in patients with COPD [4] and show that the relationship is at best a loose one. Even the largest study assessing health status by the St. George's respiratory questionnaire (SGRQ) provides only weak associations with the degree of airflow limitation, as measured by FEV_1 _[5,6]. This suggests that other factors additionally contribute to the health status in COPD. One of those may be dynamic hyperinflation, i.e. increased residual volume and total lung capacity [7], possibly as a consequence of chronic inflammation and restructuring of the airways and/or parenchyma [8,9].

The chronic inflammatory process in COPD is characterized by infiltration of the airways by neutrophils, macrophages and CD8-positive T cells [10,11]. Such features of inflammation in COPD are likely driven by various cellular pathways, including pro-inflammatory cytokines and mediators of oxidative stress [12,13]. These cytokines and mediators may not only be responsible for local airway inflammation but can also induce features of systemic inflammation in COPD [14-16]. The latter is assumed to be linked with impaired functional status in COPD [12], just as it has been shown in other chronic inflammatory conditions such as bronchiectasis, rheumatoid arthritis, chronic end-stage renal disease and inflammatory bowel syndrome [17,18]. Hence, it is not unlikely that the underlying local airway inflammation in COPD can drive impairment of health status as well [12].

We hypothesized that health status in COPD is affected by the severity of airway inflammation. The aim of our study was to test this hypothesis in a large cross-sectional study by assessing the relationship between airway inflammation, as measured by cell counts in induced sputum, and health status in COPD. In order to examine the independent effects of airway inflammation, the influence of clinical disease markers such as smoking, lung function, hyperinflation and airways hyperresponsiveness on health status was included.

*Some of the results of this study has been previously reported in the form of an abstract *[19].

## Methods

Detailed information about subjects and methodology has been published previously [20]. In brief, 114 patients with COPD were included for the Groningen Leiden Universities Chronic Obstructive Lung Disease (GLUCOLD) Study. Patients (45–75 years, current or ex-smokers ≥10 pack-years) had at least one of the following symptoms: chronic cough, sputum production, or dyspnea on exertion. Postbronchodilator forced expiratory volume in one second (FEV_1_) was > 1.3 liter and > 20% predicted and below the 90% *confidence interval *of the predicted FEV_1_[21]. Postbronchodilator FEV_1_/IVC ratio was below the 90% *confidence interval *of the predicted FEV_1_/IVC ratio. These lung function levels are compatible with GOLD stages II and III [2]. Patients were clinically stable for more than 2 months and free of common cold symptoms for 2 weeks before the measurements. They did not use a course of inhaled or oral corticosteroids during the past 3 months prior to randomization and did not have maintenance treatment with these drugs during the past 6 months. Patients with considerable co-morbidity were excluded. Usage of short-acting bronchodilators was allowed during the study. Each center's local medical ethics committee approved the protocol and patients provided written informed consent.

This study represents a cross-sectional analysis of baseline data from the GLUCOLD Study. Health status was measured using the St. George's respiratory questionnaire [22]. This is a well-validated, standardized, self-administered questionnaire, specifically designed for respiratory diseases. It contains 50 items and is divided into three sections: symptoms (distress caused by respiratory symptoms), activity (physical activities that cause or are limited by breathlessness), and impact (social and psychological effects of the disease). The total score and the three separate component scores were calculated. The scores range from zero to 100, where zero indicates best and 100 represents worst health status.

Sputum was induced and processed according to a validated technique [23]. After inhaling 200 μg salbutamol the patients inhaled hypertonic sodium chloride aerosols (4.5 w/v %) during 3 periods of 5 min. Whole sputum samples were processed within two hours from sputum induction. Differential cell counts were expressed as a percentage of nucleated cells, excluding squamous cells. A sputum sample was considered adequate when the percentage squamous cells was less than 80% [23].

Spirometry was performed, according to international guidelines [24], using the Quanjer reference values [21]. Total lung capacity (TLC) and residual volume (RV) were measured using a constant volume bodyplethysmograph [21]. Airway hyperresponsiveness was determined using the 2-minute tidal breathing method [25] and expressed as the provocative concentration causing a 20% fall in FEV_1 _(PC_20_). The diffusion capacity for carbon monoxide per liter alveolar volume (K_CO_) was measured using the single breathholding method [26]. The associations of the SGRQ total, symptom, activity and impact scores with inflammatory cell counts and various other study variables were examined using Pearson's and Spearman's rank correlation. Differences between smokers and ex-smokers were analyzed with the Student *t *test and Mann Whitney U. Skewed data (pack-years, PC_20_, % and numbers of inflammatory cells in sputum) were transformed when appropriate. Multiple linear regression analyses (ENTER method) were performed to assess the relation between health status (SGRQ total, symptom, activity and impact scores) and sputum inflammatory cell counts, independent of age, gender, current smoking, postbronchodilator FEV_1_, RV/TLC, and PC_20_. Probability values of ≤0.05 were considered significant. All analyses were performed using the Statistical Package for Social Sciences (SPSS)-12.

## Results

### Characteristics

A total of 114 patients were enrolled in the study. Patient characteristics have been published in extensive detail [20]. In short, most patients (87%) were middle-aged males (mean ± standard deviation (SD) 62% ± 8). They had a median of 41 pack-years of smoking, 37% being ex-smokers. Patients had moderate to severe COPD as based on their postbronchodilator FEV_1 _(mean ± SD 63% ± 9 of predicted (pred)) and exhibited a wide range in RV/TLC (mean ± SD 48 ± 8) and PC_20 _(geometric mean, inter quartile range (IQR) 0.6 [0.17–2.40]). A total of 110 patients adequately completed the SGRQ and 102 from these were able to produce an acceptable sputum sample. Data from the 102 patients were used for all analyses. The median SGRQ scores were indicative of moderately impaired health status (table 1). Number and differential counts of sputum cells are shown in table 2.

**Table 1:** St. George's respiratory questionnaire (SGRQ): median scores (n = 102).

	Median [IQR]
Total SGRQ score	32 [19–43]
Symptom SGRQ score	44 [34–55]
Activity SGRQ score	42 [23–54]
Impact SGRQ score	18 [8.0–30]

The SGRQ scores from 102 patients with adequate questionnaires and sputa. Data are presented as median [inter-quartile range (IQR)]. Higher SGRQ scores indicate worse health status: 0 = best, 100 = worse.

**Table 2:** Inflammatory cells in induced sputum (n = 102).

	Absolute numbers (10^4^/ml)	Percentage
Total cell count	135.0 [76.8–311.3]	-
Neutrophils	99.2 [46.7–228.6]	72.6 [59.5–82.2]
Macrophages	32.3 [17.9–61.1]	22.8 [14.8–33.3]
Eosinophils	1.4 [0.3–4.8]	1.1 [0.3–2.2]
Lymphocytes	2.1 [1.0–6.8]	1.7 [1.2–2.3]
Epithelial cells	1.3 [0.6–3.8]	1.0 [0.3–2.3]

Data are presented as median [IQR]. Total cell count refers to the total number of non-squamous cells in sputum.

### Univariate analysis

**Figure 1:**
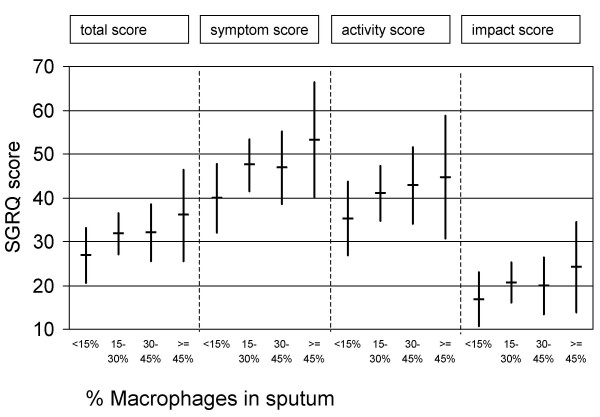
Relationship between percentages macrophages in induced sputum (*x-axis*) and SGRQ scores (*y-axis*) (n = 102). Results form linear regression analyses (B and 95% confidence interval).

The total and symptom scores were positively associated with % macrophages (r = 0.20, p = 0.050; and r = 0.20, p = 0.041, respectively). The univariate relationship between the SGRQ scores and sputum inflammatory cell counts is shown in table 3. The regression coefficient (B) in table 3 represents the strength of the association. Our results show that an increase in sputum macrophages of 1% is associated with an increase of the mean total score of 0.22 point. In addition, figure 1 shows the effect-size of a higher percentage of sputum macrophages on the SGRQ scores. Patients with <15% sputum macrophages have a mean total score of 27. The total score is on average 5 points higher in patients with 15–45% sputum macrophages, and 9 points higher in patients with > 45% sputum macrophages. A significant threshold of four units in SGRQ scores may be considered as clinically relevant [27]. The activity and impact scores were not significantly associated with sputum % macrophages (r = 0.19, p = 0.061; r_s _= 0.14, p = 0.16, respectively). No significant associations were found between all SGRQ scores and percentages of neutrophils, eosinophils, lymphocytes, epithelial cells, nor with absolute numbers of total sputum cells, neutrophils, macrophages, lymphocytes, and epithelial cells.

**Table 3:** Association between sputum cell differential counts and health status assessed with SGRQ (n = 102), results from linear regression analyses.

		SGRQ total score	symptom score	activity score	impact score
Total cell count^†^	B (95% CI)	-1.7 (-8.0 to 4.6)	-4.6 (-12 to 3.4)	-0.74 (-9.2 to 7.7)	-1.1 (-7.3 to 5.1)
% Neutrophils	B (95% CI)	-0.17 (-0.36 to 0.02)	-0.20 (-0.44 to 0.04)	-0.24 (-0.49 to 0.02)^‡‡^	-0.11 (-0.30 to 0.08)
% Macrophages	B (95% CI)	0.22 (<0.01 to 0.43)*	0.28 (0.01 to 0.56)*	0.28 (-0.01 to 0.56)^‡^	0.15 (-0.07 to 0.36)
% Eosinophils#	B (95% CI)	0.22 (-4.0–4.5)	-0.73 (-6.13–4.67)	2.42 (-3.27–8.10)	-0.61 (-4.80–3.58)

The univariate association between SGRQ scores (dependent) and sputum cell counts (independent) was expressed by regression coefficient B with corresponding 95% confidence intervals (95% CI). The regression coefficient B represents the strength of the association. Our results show that an increase in sputum macrophages of 1% is associated with an increase of the mean total score of 0.22 point, indicating that an increase in sputum macrophages of 20% is associated with an increase of the mean total score of 4.4 points, which exceeds the clinically relevant threshold of four units in SGRQ scores. ^†^Total cell count was logtransformated; # % eosinophils were transformed using the square root. *p ≤ 0.05, ^‡^p = 0.061, ^‡‡^p = 0.068.

With regard to clinical and functional parameters, the total, symptom and impact scores were higher among smokers, as compared with ex-smokers (median total score = 33.2 vs 25.8, p = 0.040; median symptom score = 46.8 vs 40.9, p < 0.01; median impact score = 19.7 vs 11.3, p = 0.023, for smokers and ex-smokers, respectively). Higher symptom scores were associated with a larger amount of pack-years (r = 0.29, p < 0.01). Higher activity scores were associated with lower postbronchodilator FEV_1 _(r = -0.24, p = 0.017), increased lung hyperinflation, as assessed by RV/TLC ratio (r = 0.25, p = 0.012) and less hyperresponsiveness, as assessed by PC_20 _(r = -0.22, p = 0.033). No associations were found between any of the SGRQ scores and CO-diffusion capacity, as assessed by KCO (data not shown).

### Multiple regression analysis

Multiple regression analysis confirmed the relationship between SGRQ total score and % sputum macrophages (B = 0.25, p = 0.021), with an explained variance of 14%. In this model there was a significant contribution of RV/TLC (B = 0.60, p = 0.002). Age, current smoking, gender, postbronchodilator FEV_1_, or PC_20 _were not significantly associated with the total score in this model. The symptom domain also remained significantly associated with % sputum macrophages in the multiple regression analysis (B = 0.30, p = 0.03), together with RV/TLC (B = 0.48, p = 0.044). Finally, multiple regression analysis showed a relationship between the activity score and % sputum macrophages (B = 0.46, p = 0.002), again with a significant contribution of RV/TLC (B = 0.61, p = 0.015) and also with PC_20 _(B = -9.3, p = 0.024).

## Discussion

This study demonstrates that health status in COPD is associated with inflammatory cell counts in induced sputum. The larger the percentage of sputum macrophages was, the more impaired a patient's health status was. This relationship was marginally modulated by the severity of hyperinflation and airways hyperresponsiveness. These findings suggest that airway inflammation independently contributes to impaired health status in COPD.

The novelty of this study is that we observed a relationship between health status and inflammatory cell counts in induced sputum in steroid naive, clinically stable patients with moderately severe COPD. In general, health status was markedly impaired, as indicated by a median SGRQ total score of 32 [5]. Interestingly, our data suggest that the inflammatory process is a stronger determinant of health status than physiological measures of hyperinflation or airflow limitation. After taking percentage sputum macrophages into account, only RV/TLC demonstrated a consistent association with the total score and subdomains of the SGRQ. This points towards an independent role of hyperinflation among the determinants of health status. Indeed, patients with a relatively high degree of hyperinflation are known to have increased breathlessness and reduced physical activities, which is even more pronounced during exercise [28,29]. This is likely to affect health status, especially with regard to the activities domain. In addition, we found some evidence of a contribution of airway hyperresponsiveness, which extends previous observations in the general population [30,31]. Although smoking is associated with health status, our results show that the relationship between health status and sputum percentages macrophages, within patients with COPD, is similar in smokers as compared with ex-smokers.

Our patient selection and methods seem to be appropriate for the current study. The sample size of 102 patients with a complete data set provided sufficient data for multivariate analysis. In general, some relationships might have been arisen by chance given the potential for multiple comparisons. However, the univariate associations between inflammatory cell counts and health status found in this study remained statistically significant when adjusted for other relevant parameters using multiple regression analysis, suggesting an independent and consistent role for inflammation with regard to health status in patients with COPD. Patients with clinically relevant co-morbidity were excluded. We reasoned that marked co-morbidity additionally affects disease-specific health status [32], which could potentially introduce confounders. We excluded all patients with maintenance therapy of inhaled corticosteroids during the last six months. Inhaled corticosteroids influence the inflammatory cell counts in induced sputum in patients with COPD [33,34], which easily might have disturbed any disease-related associations between the inflammatory process and health status.

How can we explain the observed positive association between the percentage macrophages in sputum and health status? In previous studies, neutrophils have been linked to the severity of COPD, as measured with FEV_1 _[35]. In a previous report from our study group Lapperre *et el*. categorized various functional and inflammatory features of COPD into separate complementary domains using a different statistical analysis, a so-called factor analysis. This revealed that FEV_1 _and neutrophilic inflammation are complementary dimensions that characterize patients with COPD [20]. However, several studies suggest a central role for macrophages in inflammatory processes and structural changes in the lung of patients with COPD [36,37]. Chemokines, such as monocyte chemoattractant protein 1 (MCP-1) and its receptor C-C chemokine receptor 2 (CCR2), have been implicated in the recruitment of macrophages into the bronchiolar epithelium in COPD [38]. These macrophages can release a large variety of inflammatory cytokines such as tumor necrosis factor (TNF-α), IL-8, CXC-chemokines, LTB_4_, and reactive oxygen species that are likely to drive airway inflammation in COPD. Moreover they produce elastolytic enzymes, e.g. metalloproteinases [39,40] such as macrophage elastase (MME), that may degrade the extracellular matrix and thus contribute to the development of parenchymal damage and thereby to pulmonary emphysema in COPD [13,36,41].

The novelty of this study is that associations were observed between health status and local airways inflammation, whilst previous studies suggested associations between impaired health status and systemic inflammation in COPD [14,42]. Previously, it has been suggested that the systemic inflammatory response may be due to a overflow of pulmonary mediators from the airways [14]. However, Vernooy *et al*. showed that soluble tumour necrosis factor receptor (sTNF-R) and IL8 in sputum and plasma were not correlated, suggesting that the inflammatory process in the local and systemic compartment are regulated differentially [43,44]. In the airways neutrophilic inflammation is associated with lower FEV_1 _levels in COPD [45]. The role of airway macrophages may be linked to different pathophysiological processes as mentioned above. Environmental exposures such as tobacco smoke may promote macrophage-induced alveolar damage [46], leading to impaired alveolar-capillary gastransport and accompanying changes in health status. Interestingly, our results are suggestive of a distinct role for the differential cell counts rather than the total amount of macrophages. Taken together, we may speculate that the local and systemic inflammatory responses are partly differentially regulated, mutually determining the COPD phenotype. If so, this will be of major importance when developing effective interventions in this disease.

The percentage macrophages in sputum was associated with the SGRQ total score (a summary measure of health status), as well as the SGRQ symptom score (severity of symptoms) and SGRQ activity score (physical activities that cause or are limited by breathlessness). As shown in figure 1, the differences in health status between patients with relatively higher and lower percentages of macrophages can be considered as clinically relevant, because they reached the clinically significant threshold of four units in SGRQ scores [27]. This suggests that airway inflammation in COPD is relevant for disease outcome in daily life. Inflammatory cell counts in sputum were not associated with the impact score. This score measures social and psychological effects of the disease, such as anxiety and coping, and it is plausible that this score is less influenced by the inflammatory component of the disease. It is important to notice that only a limited part of health status could be explained by the severity of airway inflammation. The likely reason for this is that a wide spectrum of disease processes potentially affects health status [4]. Furthermore, other factors such as coping or the presence and frequency of exacerbations might also play an additional role in its impairment in patients with COPD [47].

We observed a consistent and independent contribution of hyperinflation on health status in patients with mild to moderate COPD. This is in line with a previous study, where hyperinflation was associated with poor health status in very severe patients with COPD who were using long-term oxygen treatment [48]. Hyperinflation causes an increase in lung volume with a concomitant increase of work of breathing, functional impairment of inspiratory muscle function, and adverse effects on haemodynamics which all may contribute to dyspnea [49]. In a recent study in COPD tiotropium bromide significantly decreased the residual volume [7], which was correlated with a decrease in dyspnea. This is indicative of the clinical relevance of hyperinflation in COPD, and the more so because dyspnea appears to be an important factor influencing health status [50]. In addition, the activity score measures physical activities that on the one hand induce breathlessness, and on the other may become limited by this particular symptom. Therefore, the current associations between hyperinflation and various domains of health status are not unexpected.

In conclusion, we have observed that a worse health status in COPD is significantly associated with higher inflammatory cell counts in induced sputum, whereas only marginally additional contributions were found for lung function measures reflecting hyperinflation. Our observation that airway inflammation negatively affects health status of COPD patients may have clinical relevance. At present, anti-inflammatory therapy with inhaled corticosteroids is a recommended treatment option in patients with advanced COPD [2]. This has been shown to reduce deterioration in health status [5,51]. If health status is partly driven by the local inflammatory process in COPD this may provide a rationale for the usage of anti-inflammatory therapy in COPD. It now needs to be examined whether the severity of airway inflammation predicts the benefits of long-term anti-inflammatory intervention on health status in COPD.

## Declaration of competing interest

The author(s) declare that they have no competing interests.

## Authors' contributions

JS carried out measurements, coordinated the study, performed statistical analyses and drafted the document; DP participated in the design of the study and coordination and helped drafting of the manuscript; TL carried out measurements, coordinated the study and helped drafting of the manuscript; MG carried out measurements, coordinated the study and helped drafting of the manuscript; HT and HK participated in the design of the study and coordination and helped drafting of the manuscript; JKS participated in the design of the study and performed statistical analyses; DJ participated in the design of the study and performed statistical analyses; PS participated in the design of the study and coordination and helped drafting of the manuscript. All contributors approved the final manuscript.
